# Catheter Ablation of Atrial Fibrillation in Patients With Functional Mitral Regurgitation and Left Ventricular Systolic Dysfunction

**DOI:** 10.3389/fcvm.2020.596491

**Published:** 2020-12-14

**Authors:** Jin-Tao Wu, Junaid A. B. Zaman, H. Yakup Yakupoglu, Boyalla Vennela, Cantor Emily, Karim Nabeela, Julian Jarman, Shouvik Haldar, David Gareth Jones, Hussain Wajid, Rui Shi, Zhong Chen, Vias Markides, Tom Wong

**Affiliations:** ^1^Heart Centre of Henan Provincial People's Hospital, Central China Fuwai Hospital, Central China Fuwai Hospital of Zhengzhou University, Zhengzhou, China; ^2^Heart Rhythm Centre, The Royal Brompton and Harefield National Health Service (NHS) Foundation Trust, National Heart and Lung Institute, Imperial College London, London, United Kingdom; ^3^Echocardiography Department, The Royal Brompton and Harefield National Health Service (NHS) Foundation Trust, National Heart and Lung Institute, Imperial College London, London, United Kingdom

**Keywords:** atrial fibrillation, catheter ablation, functional mitral regurgitation, heart rhythm, systolic dysfunction

## Abstract

**Background:** The efficacy of catheter ablation for atrial fibrillation (AF) in patients with functional mitral regurgitation (MR) and left ventricular (LV) systolic dysfunction (LVSD) is not known. The aim of the study is to determine the efficacy of catheter ablation for AF in patients with functional MR and LVSD, and to validate its effects on the severity of MR and cardiac reverse remodeling.

**Methods:** We performed a retrospective study of 54 patients with functional MR who underwent AF ablation, including 21 (38.9%) with LVSD and 33 (61.1%) with normal LV systolic function (LVF). The primary outcomes evaluated were freedom from recurrent atrial tachyarrhythmia (ATa), severity of MR, and left atrial (LA) and LV remodeling.

**Results:** During a mean follow-up of 20.7 ± 16.8 months, freedom from recurrent ATa was not significantly different between patients with LVSD and those with normal LVF after the first ablation (*P* = 0.301) and after multiple ablations (*P* = 0.728). Multivariable predictors of recurrent ATa were AF duration [hazard ratio (HR) 1.12, 95% confidence interval (CI) 1.01–1.25; *P* = 0.039), previous stroke (HR 5.28, 95% CI 1.46–19.14; *P* = 0.011), and estimated glomerular filtration rate (HR 0.97, 95% CI 0.95–0.99; *P* = 0.012). Compared with baseline, there was a significant reduction in severity of MR (*P* = 0.007), LA size (*P* < 0.001) and LV end-systolic dimension (*P* = 0.008), and improvement in the LV ejection fraction (*P* = 0.001) after restoring sinus rhythm in patients with LVSD.

**Conclusion:** Catheter ablation is a valid option for the treatment of AF in patients with functional MR and LVSD, even though multiple procedures may be required.

## Introduction

Atrial fibrillation (AF) and functional mitral regurgitation (MR) are common cardiac disorders, which are associated with increased cardiovascular mortality and hospitalization rates. These two disorders frequently coexist and promote each other (1). Currently, for patients with AF and functional MR, the optimal therapy is unclear. Simultaneous treatment of AF and MR is ideal. However, surgical therapy is not recommended for treatment of isolated refractory AF (2). Additionally, many patients with functional MR are not referred for mitral valve surgery because of a high surgical risk or comorbidities and a lack of proven mortality benefit (3). Transcatheter mitral valve repair in patients with functional MR is currently feasible in many countries worldwide. However, a survival benefit of this technique compared with optimal medical therapy according to current guidelines (4) has not yet been proven. Furthermore, a recent study showed that pre-existing AF was associated with worse clinical outcomes in patients who underwent transcatheter mitral valve repair (5).

Among patients with AF and functional MR, some have normal left ventricular (LV) systolic function, in which functional MR develops as a result of left atrial (LA) enlargement. This results in a dilated mitral annulus and reduced leaflet coaptation, and is known as atrial functional MR (1, 6). However, the majority of these patients have left ventricular systolic dysfunction (LVSD) due to loss of atrioventricular synchrony caused by AF (7) or pre-existing ventricular cardiomyopathy, resulting in subsequent functional MR and AF. Catheter ablation of AF in patients with functional MR and normal LV systolic function appears to have good efficacy and improves the severity of MR (1, 8). That said, whether outcomes following AF ablation are similar in patients with functional MR and LVSD to those with functional MR and normal LV systolic function is unclear. Therefore, the present study aimed to determine the efficacy of catheter ablation for AF in patients with functional MR and LVSD, and to validate its effects on the severity of MR and cardiac reverse remodeling.

## Materials and Methods

### Study Population

Consecutive patients with AF who were admitted to the Royal Brompton and Harefield NHS foundation Trust for first catheter ablation of AF between 2013 and 2018 were retrospectively reviewed. Reports from transthoracic echocardiograms that were performed before catheter ablation of AF were screened. MR was defined as functional if leaflets showed normal morphology, but did not properly coapt because of either LV or left atrial dilatation (9). Functional MR was classified as either absent (Grade 0) or as one of the four progressive degrees of severity from mild (Grade 1), mild to moderate (Grade 2), moderate to severe (Grade 3), and severe (Grade 4) MR (9). All patients with significant functional MR (MR ≥ Grade 2) were enrolled in this study. We excluded patients with primary MR (mitral valve prolapse, rheumatic disease, endocarditis, previous papillary muscle rupture, or abnormalities in mitral valve leaflets or chordae), patients with a history of mitral valve replacement or mitral valve repair (surgical or transcatheter) and aortic valve replacement (surgical or transcatheter), patients with congenital heart disease, patients with missing echocardiographic data. Patients with a baseline left ventricular ejection fraction (LVEF) < 50% were designated as having LVSD and those with an LVEF ≥ 50% as having normal LV systolic function (10). Chronic kidney disease was defined by an estimated glomerular filtration rate (eGFR) of <60 mL/min. The study complied with the Declaration of Helsinki and the study protocol was approved by the Research and Development Department at the Royal Brompton and Harefield NHS Foundation Trust.

### Ablation Procedure

After obtaining written informed consent, the procedures were performed under general anesthesia with on-table transesophageal echo to guide subsequent trans-septal puncture and exclude intracardiac thrombus. Warfarin was continued and direct oral anticoagulants were minimally interrupted. Intravenous heparin was administered during the procedure and doses were adjusted to achieve an activation clotting time of >300 ms.

The CARTO 3-dimensional electroanatomical mapping system (Biosense Webster, Diamond Bar, CA, USA) was used in the majority of procedures. Ablation techniques varied according to the operator's discretion, anatomical features, type of AF/atrial tachycardia (AT), and history of previous ablations. Techniques included ipsilateral pulmonary vein isolation (PVI) with a wide area of circumferential ablation, focal activity ablations, superior vena cava isolation, and atrial substrate modification by applying ablation at complex fractionated atrial electrograms (CFAEs), the cavotricuspid isthmus (CTI), and/or additional LA linear ablation, such as the roof line, posterior box lesion, or mitral valve line from the annulus to the inferior pulmonary vein (PV). During PVI, a circumferential mapping catheter (Lasso, Biosense Webster, CA, USA) was placed inside the ipsilateral PV. The endpoint of PVI was defined as the absence of any PV spike potential recorded on the Lasso catheter. In AT procedures, tachycardia was carefully mapped and re-entry circuits or the origin of focal ATs was targeted for ablation. If the LA was entered, PVs were also checked and isolated if a conduction gap was present.

### Transthoracic Echocardiography

Standard 2-dimensional Doppler echocardiography with color flow mapping was performed preoperatively in all patients. Severity of MR was semi-quantitatively assessed on a scale from 1 to 4 according to the quantitative measure of the effective regurgitant orifice area and regurgitant volume by using the proximal isovelocity surface area method (9). In cases with an extremely eccentric jet, the vena contracta width was measured in the parasternal long-axis view and measured using the zoom mode at the narrowest portion of the regurgitant jet (9). Left atrial volume (LAV) was obtained using the biplane method of disks (11) and indexed to body surface area. LV diameters were determined from parasternal long-axis acquisitions (11). The LVEF was calculated by the modified biplane Simpson's method (11). Subsequent echocardiographic follow-up was performed in our institution or in the patient's home institution. The echocardiograms data were screened independently by two observers who were blinded to the patient details, and any differences between the observers were resolved by consensus.

### Follow-Up

Antiarrhythmic drugs were prescribed at discharge for specific indications (e.g., persistent AF, requirement for cardioversion, large LA size) and at the operator's discretion. Patients were followed up with 48 or 72-h Holter monitoring at 3, 6, and 12 months, and on an annual basis beyond 1 year after the ablation procedure. Reported symptoms outside these time points were assessed with a 12-lead electrocardiogram (ECG) and further 48-h Holter as indicated. Device checks were scheduled every 6–12 months or more frequent if necessary. Twelve-lead ECG, Holter recordings, and device-based electrograms during follow-up were reviewed. The primary endpoint was defined as recurrence of confirmed atrial tachyarrhythmia (ATa) lasting longer than 30 s (documented by ECG, Holter recordings or device-based electrograms) after a 3-month blanking period after catheter ablation. Echocardiograms were performed at different time points after the first ablation at the treating physician's discretion. Recurrence at the time of follow-up echocardiography was defined as any electrocardiographic recurrence during 3 months preceding the echocardiogram.

### Statistical Analysis

All analyses were performed using SPSS software version 17.0 (SPSS Inc., Chicago, IL, USA). Continuous data are presented as mean ± standard deviation and were compared using the unpaired independent-samples *t*-test or paired samples *t*-test. Categorical variables are presented as numbers and percentages of the group total and were compared using the χ^2^ test or Fisher's exact test as appropriate. Differences between non-normally distributed and ordinal variables were tested with the Wilcoxon signed-ranks test or the Mann–Whitney U-test for paired and unpaired data, respectively. The Kaplan–Meier method was used for survival analysis after the first and multiple ablations and *P*-values were calculated with the log-rank test. Univariable cox proportional hazards regression analysis was used to identify clinical and demographic variables associated with recurrence of arrhythmia during follow-up. Variables that showed *P* < 0.10 in univariable analysis were included in the multivariable models (forward likelihood ratio). All probability values were 2-sided and values of *P* < 0.05 were considered statistically significant.

## Results

### Baseline Characteristics

A total of 1,872 patients with AF were screened between 2013 and 2018 and 59 patients with AF and functional MR were initially enrolled. Of the 59 patients, five patients without follow-up data were excluded and the remaining 54 (91.5%) were included in the final study sample. Patients with LVSD comprised 38.9% (21/54), while 61.1% (33/54) were patients with normal LV systolic function. The etiology of LVSD was ischaemic cardiomyopathy in 9.5% (2/21) of patients, and all other patients had a nonischaemic etiology. The clinical characteristics are summarized in Table 1. Patients with LVSD were younger (*P* = 0.003), and more likely to be men (*P* = 0.010), have a larger LAV (*P* = 0.025), and have larger LV end-systolic (*P* < 0.001) and end-diastolic dimensions (*P* < 0.001)compared with those with normal LV systolic function.

**Table 1:** Baseline characteristics of the study population.

**Variables**	**All, *n* = 54**	**LVSD, *n* = 21**	**Normal LVF, *n* = 33**	***P*-value**
Age, years	67 ± 10	61 ± 12	70 ± 7	0.003
Female, *n* (%)	16 (29.6%)	2 (9.5%)	14 (42.4%)	0.010
Body mass index, kg/m^2^	28.6 ± 5.2	29.1 ± 4.9	28.4 ± 5.4	0.640
AF duration, years	3.1 ± 2.8	2.7 ± 2.8	3.4 ± 2.7	0.397
Paroxysmal AF, *n* (%)	9 (16.7%)	1 (4.8%)	8 (24.2%)	0.075
Persistent AF, *n* (%)	45 (83.3%)	20 (95.2%)	25 (75.8%)	
Previous ablation (non-AF), *n* (%)	3 (5.6%)	1 (4.8%)	2 (6.1%)	1.000
Previous CABG, *n* (%)	3 (5.6%)	1 (4.8%)	2 (6.1%)	1.000
PM/ICD, *n* (%)	7 (13.0%)	3 (14.3%)	4 (12.1%)	1.000
CRT, *n* (%)	3 (5.6%)	3 (14.3%)	0 (0%)	0.054
Watchman device, *n* (%)	2 (3.7%)	1 (4.8%)	1 (3.0%)	1.000
CHA_2_DS_2_-VASc score	2.5 ± 1.2	2.4 ± 1.4	2.6 ± 1.1	0.663
Diabetes mellitus, *n* (%)	6 (11.1%)	3 (14.3%)	3 (9.1%)	0.667
Hypertension, *n* (%)	24 (44.4%)	8 (38.1%)	16 (48.5%)	0.454
Previous stroke or TIA, *n* (%)	3 (5.6%)	2 (9.5%)	1 (3.0%)	0.553
Congestive heart failure, *n* (%)	20 (37.0%)	18 (85.7%)	2 (6.1%)	<0.001
Coronary artery disease, *n* (%)	12 (22.2%)	4 (19.0%)	8 (24.2%)	0.747
Chronic kidney disease, *n* (%)	17 (31.5%)	6 (28.6%)	11 (33.3%)	0.713
eGFR, mL/min	65.5 ± 17.8	67.0 ± 17.9	64.6 ± 18.0	0.631
**Antiarrhythmic drugs on discharge**, ***n*** **(%)**				
None	9 (16.7%)	2 (9.5%)	7 (21.2%)	0.456
Class I or III	19 (35.2%)	6 (28.6%)	13 (39.4%)	0.417
Beta-blocker	39 (72.2%)	17 (81.0%)	22 (66.7%)	0.253
Calcium channel blocker	4 (7.4%)	1 (4.8%)	3 (9.1%)	1.000
MR grade, *n* (%)				0.061
Grade 2	43 (79.6%)	14 (66.7%)	29 (87.9%)	
Grade 3	8 (14.8%)	5 (23.8%)	3 (9.1%)	
Grade 4	3 (5.6%)	2 (9.5)	1 (3.0%)	
LVEF, %	49.7 ± 15.4	32.1 ± 7.5	60.9 ± 4.8	<0.001
LA volume, mL	104.0 ± 32.2	116.2 ± 29.7	96.3 ± 31.7	0.025
LA volume index, mL/m^2^	52.8 ± 14.5	56.7 ± 14.9	50.3 ± 14.0	0.119
LVEDD, cm	5.3 ± 0.8	6.0 ± 0.7	4.5 ± 0.9	<0.001
LVESD, cm	4.0 ± 1.1	5.0 ± 0.8	3.3 ± 0.5	<0.001

*LVSD, left ventricular systolic dysfunction; LVF, left ventricular function; AF, atrial fibrillation; CABG, coronary artery bypass grafting; PM, pacemaker; ICD, implantable cardioverter-defibrillator; CRT, cardiac resynchronization therapy; eGFR, estimated glomerular filtration rate; MR, mitral regurgitation; LVEF, left ventricular ejection fraction; LA, left atrial; LVEDD, left ventricular end-diastolic dimensions; LVESD, left ventricular end-systolic dimensions*.

### Procedural and Midterm Efficacy Outcomes

A total of 82 AF or AT (post-AF ablation) procedures were performed during the study period, and one patient underwent up to a maximum of four procedures. The details of the ablation procedures are shown in Table 2. At the first procedure, all patients underwent successful PVI ablation, and a CTI line was created in 13 (61.9%) patients with LVSD and in 12 (36.4%) patients with normal LV function (*P* = 0.067). CFAEs were also ablated during the first procedure in 9 (42.9%) patients with LVSD and in 13 (39.4%) with normal LV function, with no significant difference between the groups. More patients with LVSD tended to have additional LA linear ablation than those with normal LV function (66.7 vs. 39.4%, *P* = 0.051). A total of 21 (38.9%) patients underwent multiple procedures. One patient was complicated by a Lasso catheter caught in chordae tendineae of mitral valve in his second ablation. This catheter eventually became free, but there was development of moderate-severe MR. Therefore, this patient was only included in the follow-up analysis of the first procedure.

**Table 2:** Details of the ablation procedures.

**Variables**	**All, *n* = 54**	**LVSD, *n* = 21**	**Normal LVF, *n* = 33**
Mean *n* of procedures	1.5 ± 0.7	1.4 ± 0.6	1.6 ± 0.8
**1st procedure**	54	21	33
PV isolation, *n* (%)	54 (100%)	21 (100%)	33 (100%)
Cavotricuspid isthmus ablation, *n* (%)	25 (46.3%)	13 (61.9%)	12 (36.4%)
Additional LA lines ablation, *n* (%)	27 (50.0%)	14 (66.7%)	13 (39.4%)
CFAE ablation, *n* (%)	22 (40.7%)	9 (42.9%)	13 (39.4%)
AT ablation, *n* (%)	5 (9.3%)	2 (9.5%)	3 (9.1%)
**2nd procedure**	21	8	13
PV re-isolation, *n* (%)	13 (61.9%)	4 (50.0%)	9 (69.2%)
Cavotricuspid isthmus ablation, *n* (%)	5 (23.8%)	2 (25.0%)	3 (23.1%)
Additional LA lines ablation, *n* (%)	15 (71.4%)	6 (75.0%)	9 (69.2%)
CFAE ablation, *n* (%)	7 (33.3%)	2 (25.0%)	5 (38.5%)
AT ablation, *n* (%)	7 (33.3%)	3 (37.5%)	4 (30.8%)
**3rd procedure**	6	1	5
PV re-isolation, *n* (%)	2 (33.3%)	0 (0%)	2 (40.0%)
Cavotricuspid isthmus ablation, *n* (%)	1 (16.7%)	0 (0%)	1 (20.0%)
Additional LA lines ablation, *n* (%)	3 (50.0%)	1 (100%)	2 (40.0%)
CFAE ablation, *n* (%)	4 (66.7%)	0 (0%)	4 (80.0%)
AT ablation, *n* (%)	2 (33.3%)	1 (100%)	1 (20.0%)
SVC isolation, *n* (%)	1 (16.7%)	0 (0%)	1 (20.0%)
**4th procedure**	1	0	1
Additional LA lines ablation, *n* (%)	1 (100%)	0 (0%)	1 (100%)
AT ablation, *n* (%)	1 (100%)	0 (0%)	1 (100%)

*LVSD, left ventricular systolic dysfunction; LVF, left ventricular function; PV, pulmonary vein; LA, left atrial; CFAE, complex fractionated atrial electrograms; AT, atrial tachycardia; SVC, superior vena cava*.

**Figure 1:**
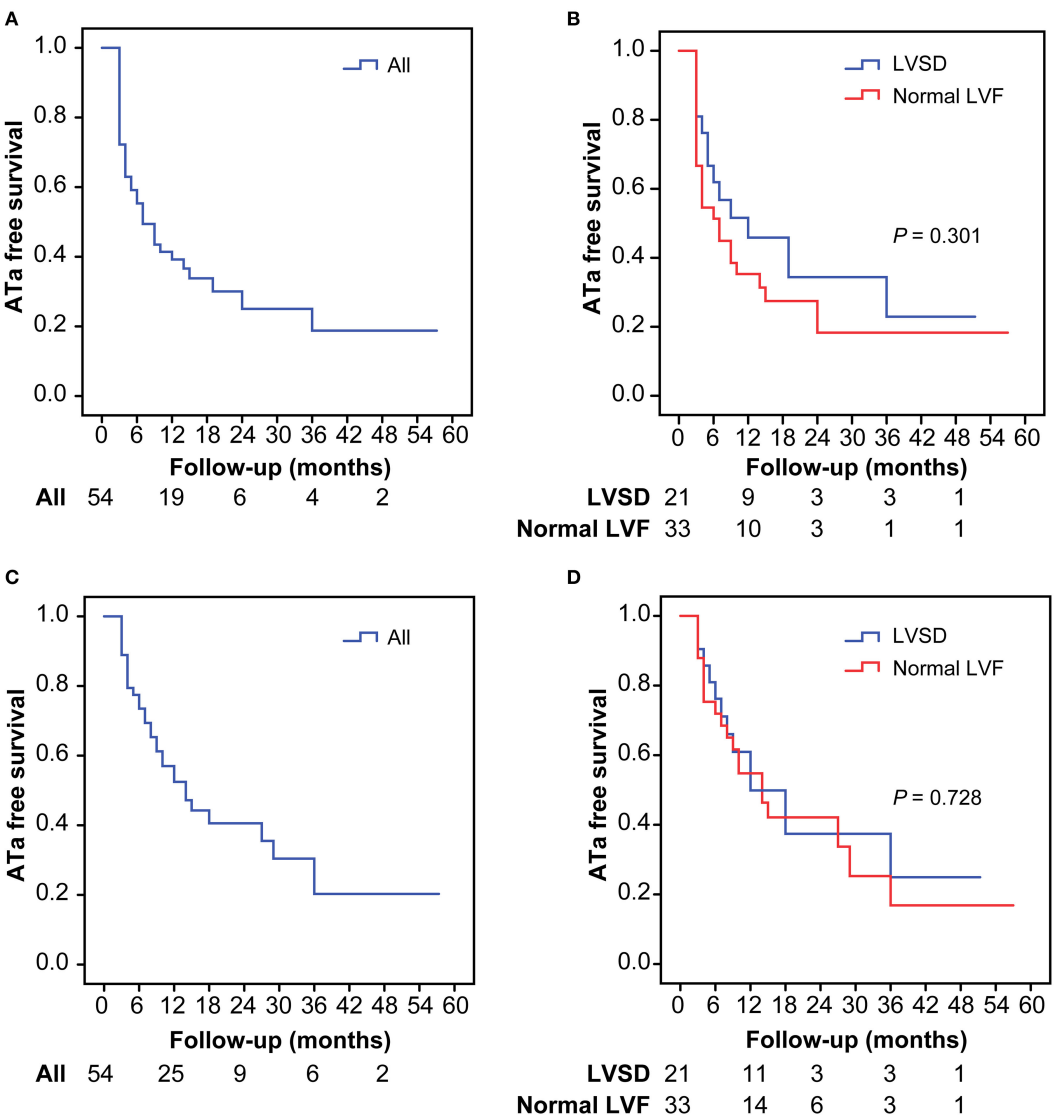
Kaplan–Meier analysis for atrial tachyarrhythmia-free survival after the first procedures in the overall population **(A)** and in patients with LVSD and normal LVF **(B)**, after multiple procedures in the overall population **(C)**, and in patients with LVSD and normal LVF **(D)**. ATa, atrial tachyarrhythmia; LVSD, left ventricular systolic dysfunction; LVF, left ventricular function.

At 12 months, freedom from recurrent ATa was not significantly different between patients with LVSD and those with normal LV function after the first ablation (45.9 vs. 35.3%, *P* = 0.113) and after multiple ablations (49.9 vs. 54.8%, *P* = 0.479). Freedom from recurrent ATa at 12 months for the overall cohort after the first ablation was 39.2% and that after multiple ablations was 52.4% (Figures 1A,C). During a mean follow-up of 20.7 ± 16.8 months (range, 3–68 months), freedom from recurrent ATa was also not significantly different between patients with LVSD and those with normal LV function after the first ablation (*P* =0.301) and after multiple ablations (*P* =0.728) (Figures 1B,D).

### Predictors of Recurrence of AF

Univariable Cox proportional hazards regression analysis showed that for patients with functional MR after the first ablation, the duration of AF [hazard ratio (HR) 1.13, 95% confidence interval (CI) 1.02–1.26; *P* = 0.022], the CHA_2_DS_2_-VASc score (HR 1.37, 95% CI 1.04–1.82; *P* = 0.027), a CHA_2_DS_2_-VASc score >2 (HR 2.05, 95% CI 1.06–3.98; *P* = 0.033), previous stroke (HR 4.25, 95% CI 1.20–15.06; *P* = 0.025), eGFR (HR 0.98, 95% CI 0.96–1.00; *P* = 0.013), and LAV (HR 1.01, 95% CI 1.00–1.02; *P* = 0.040) were associated with the risk of recurrence (Table 3). In multivariate analysis, the duration of AF (HR 1.12, 95% CI 1.01–1.25; *P* = 0.039), previous stroke (HR 5.28, 95% CI 1.46–19.14; *P* = 0.011), and eGFR (HR 0.97, 95% CI 0.95–0.99; *P* = 0.012) were independent predictors of recurrence (Table 3).

**Table 3:** Univariate and multivariate predictors of recurrence of atrial fibrillation after the first ablation procedure.

**Variable**	**Univariate Cox Regression**			**Multivariate Cox Regression**		
	**HR**	**95% CI**	***P-*value**	**HR**	**95% CI**	***P*-value**
Age	1.02	0.98–1.05	0.329			
Female	0.71	0.33–1.50	0.367			
Body mass index	1.01	0.95–1.08	0.678			
AF duration	1.13	1.02–1.26	0.022	1.12	1.01–1.25	0.039
Persistent AF	1.92	0.74–4.98	0.181			
PM/ICD	1.22	0.47–3.15	0.685			
CRT	1.68	0.51–5.49	0.393			
CHA_2_DS_2_-VASc score	1.37	1.04–1.82	0.027			
CHA_2_DS_2_-VASc >2	2.05	1.06–3.98	0.033			
Diabetes mellitus	1.32	0.55–3.16	0.541			
Hypertension	1.37	0.72–2.62	0.341			
Previous stroke	4.25	1.20–15.06	0.025	5.28	1.46–19.14	0.011
Congestive heart failure	0.72	0.33–1.58	0.411			
Coronary artery disease	1.82	0.89–3.72	0.100			
eGFR	0.98	0.96–1.00	0.013	0.97	0.95–0.99	0.012
LVEF	1.01	0.99–1.03	0.496			
LVEF <50	0.72	0.36–1.42	0.338			
LA volume	1.01	1.00–1.02	0.040			
LA volume index	1.02	1.00–1.04	0.089			
LVEDD	0.91	0.61–1.35	0.634			
LVESD	0.89	0.65–1.21	0.442			
Additional LA lines ablation	1.19	0.62–2.27	0.603			
Cavotricuspid isthmus ablation	0.66	0.34–1.27	0.212			
CFAE Ablation	1.22	0.63–2.36	0.562			

*AF, atrial fibrillation; PM, pacemaker; ICD, implantable cardioverter-defibrillator; CRT, cardiac resynchronization therapy; eGFR, estimated glomerular filtration rate; LVEF, left ventricular ejection fraction; LA, left atrial; LVEDD, left ventricular end-diastolic dimensions; LVESD, left ventricular end-systolic dimensions; CFAE, complex fractionated atrial electrograms; HR, hazard ratio; CI, confidence interval*.

### Effect on MR and Cardiac Remodeling

Follow-up echocardiograms were available in 41 of the 54 patients at a mean of 10.8 ± 7.6 months after the initial procedure. A total of 18 of these were patients with LVSD and 23 patients had normal LV systolic function. Rhythm status at follow-up was defined as described above, and only applied to the 3 months preceding echocardiography. Using this definition, nine patients with LVSD were in sinus rhythm and nine in had recurrence of AF after the first ablation. Ten patients with normal LV function were in sinus rhythm and 13 had recurrence of AF.

**Figure 2:**
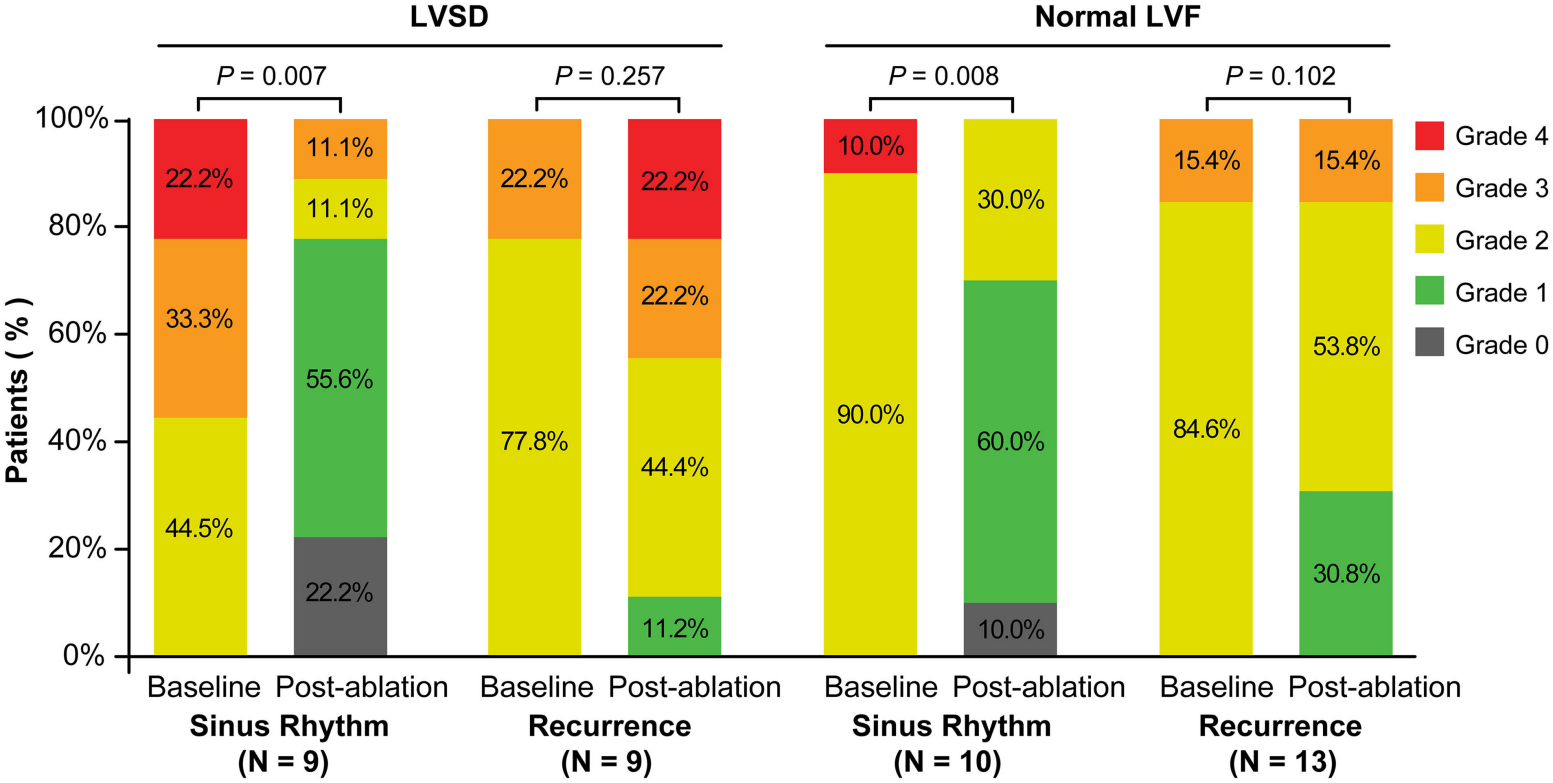
Severity of MR at baseline and post-ablation according to recurrence of atrial fibrillation or sinus rhythm categorized by the rhythm at the time of follow-up in patents with LVSD and normal LVF. LVSD, left ventricular systolic dysfunction; LVF, left ventricular function.

**Figure 3:**
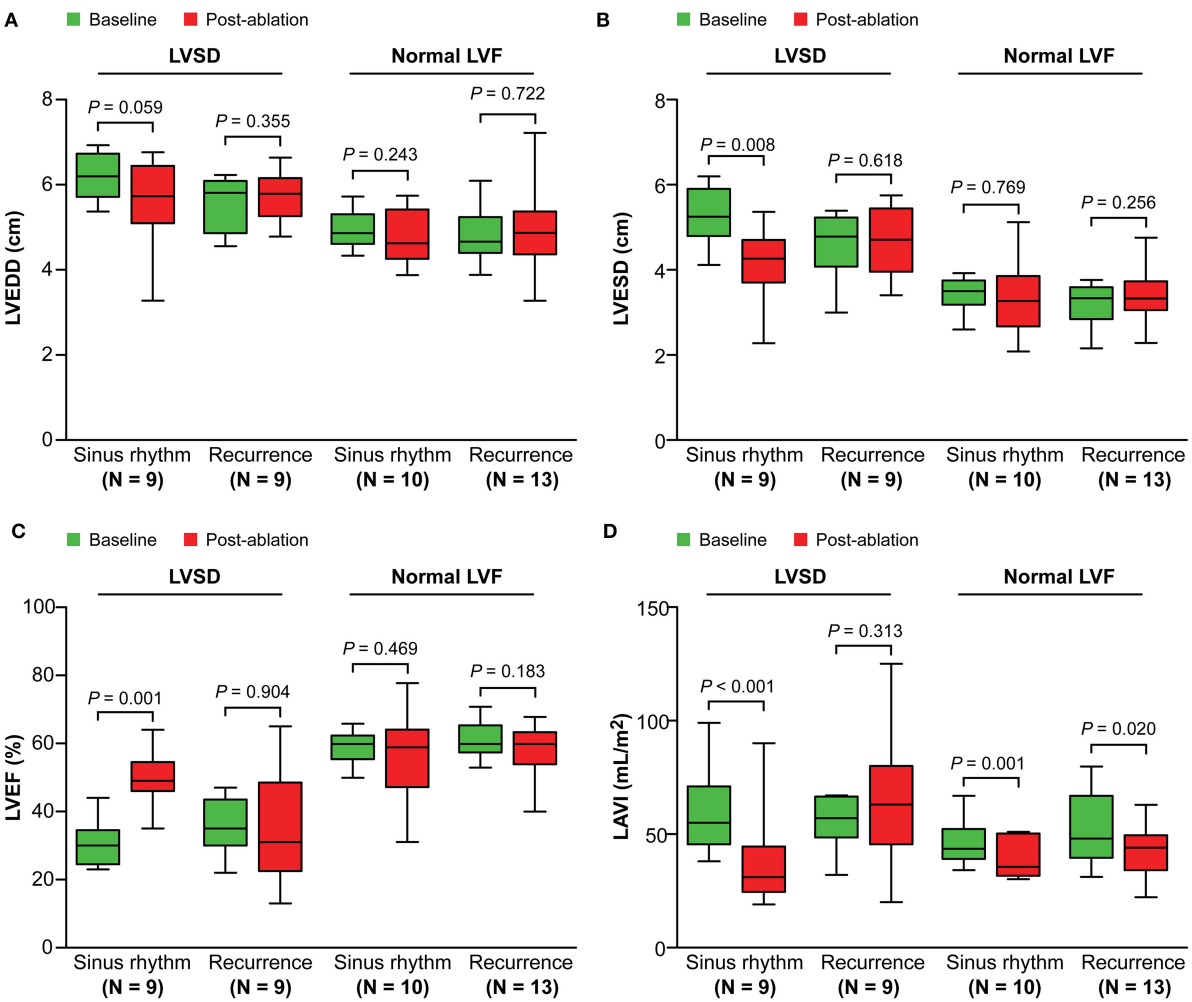
Change in left ventricular dimensions **(A,B)**, the left ventricular ejection fraction **(C)**, and the left atrial volume index **(D)** from baseline to post-ablation. LVSD, left ventricular systolic dysfunction; LVF, left ventricular function; LVEDD, left ventricular end-diastolic dimensions; LVESD, left ventricular end-systolic dimensions; LVEF, left ventricular ejection fraction; LAVI, left atrial volume index.

In patients with LVSD, patients in sinus rhythm showed a significant decrease in severity of MR (*P* = 0.007), LAV index (*P* < 0.001), and LV end-systolic dimension (*P* = 0.008), and improvement in the LVEF (*P* = 0.001), compared with baseline (Figures 2, 3). Similarly, in patients with normal LV function, patients in sinus rhythm showed a significant decrease in severity of MR (*P* = 0.008) and the LAV index (*P* = 0.001) (Figures 2, 3).

## Discussion

The main findings in this study were as follows. (1) AF ablation is feasible in patients with functional MR and LVSD, but recurrence rates are high and multiple procedures may be required. (2) The duration of AF, eGFR, and previous stroke were associated with worse results of AF ablation in patients with functional MR. (3) Freedom from ATa is associated with a reduction in severity of MR and positive LA and LV remodeling in patients with LVSD.

### Comparison With Previous Studies

Several studies have examined the outcome of catheter ablation of AF in patients with MR. Gertz et al. (1) retrospectively compared 53 patients with moderate to severe functional MR and normal LV systolic function (LVEF ≥ 50%) with a matched AF cohort with trivial and/or mild MR during first AF ablation. These authors found that patients with successful ablations showed a significant reduction in severity of MR and LA size. A subsequent case report also described improvement in a reduction in severity of MR after catheter ablation for AF in a patient with severe functional MR and normal LV systolic function (8). Another study compared the outcome of catheter ablation in patients with paroxysmal AF with significant primary or functional MR (12). This previous study showed that improvement of severity of MR was more remarkable in patients with functional MR compared with those with primary MR. There was also a tendency toward a lower rate of recurrent atrial arrhythmia in patients with functional MR than in those with primary MR. These findings were found in another study conducted by Zhao et al. (13). Additionally, Gertz et al. (14) also evaluated the effect of MR on recurrence rates after catheter ablation of AF, and found that LA size, but not MR, was an independent predictor of recurrence of AF. In contrast, another study of 216 patients with long-standing persistent AF who underwent catheter ablation showed that MR, as well as LA size, were independent predictors of recurrence of AF (13). Notably, both of these outcome studies included patients with functional MR and primary MR. To the best of our knowledge, no study has investigated the outcome of catheter ablation of AF in the subgroup of patients presenting with functional MR and LVSD.

### Ablation Efficacy and Predictors of Recurrence of Arrhythmia

In this comparative study of AF ablation in patients with functional MR, we observed no difference in outcomes between those with and those without LVSD. A previous single-center cohort study by Black-Maier et al. (15) showed no difference in ablation outcomes between patients with heart failure with a reduced LVEF and those with a preserved LVEF. In Black-Maier et al.'s study, the study population was patients with heart failure and only 13% (31/230) of the study population had MR. The current study investigated a population with functional MR and LVSD, which is different from that of Black-Maier et al.'s study. In the present study, we also showed that the duration of AF, eGFR, and previous stroke were independent predictors of recurrence of AF. LA size is a well-known predictor of recurrence of AF after catheter ablation (16). However, in the present study, although LA size was a predictor of recurrence of AF in univariate analysis, it was not an independent predictor in multivariate analysis. This may be explained by the findings of a previous study (17), which showed that while patients with a severely enlarged left atrium may be accurately identified as “high risk” for AF recurrence, patients with mild-to-moderate LA enlargement exhibit varying responses to catheter ablation.

### MR and Cardiac Remodeling After AF Ablation

In the present study, we found that freedom from recurrent ATa after ablation was associated with a reduction in severity of MR and LA size in patients with normal LV systolic function. This finding is in accordance with findings of a previous study by Gertz et al. (1). Our study also showed a reduction in severity of MR and LA size in patients with LVSD. Moreover, positive LV remodeling occurred in patients with LVSD. Our findings suggest that, similar to patients with AF with functional MR and normal LV function, patients with AF with functional MR and LVSD still benefit from restoration of sinus rhythm by catheter ablation of AF. In our study, the severity of MR and cardiac remodeling were evaluated during follow-up under the condition of maintaining rhythm status for at least 3 months. A recent study showed that there was significant improvement in LV function after sinus rhythm restoration for ≥3 days by electrical cardioversion in patients with idiopathic cardiomyopathy and AF (18). Therefore, a reduction in severity of MR and positive cardiac reverse remodeling in our study population may have occurred in an earlier stage after restoration of sinus rhythm. Furthermore, another previous study conducted by Zhao et al. (19) examined long-term outcomes of catheter ablation of AF in dilated cardiomyopathy. These authors showed that freedom from ATa was associated with improved LV systolic function during, but not beyond, 3 years after ablation, likely due to unstoppable progression of cardiomyopathy. Therefore, further studies of long-term follow up are required for our study population.

## Limitations

Several limitations of our study should be considered. First, a small sample size is a major limitation of this study and this may have introduced statistical bias. Further studies with larger sample sizes are needed to further evaluate the role of catheter ablation for AF in patients with functional MR and LVSD. Second, the precise LVEF cutoff regarding the definition for LVSD widely varies in the literature (10, 20). Since normal LV systolic function in previous studies on atrial functional MR was usually defined as an LVEF ≥ 50% (1), we defined LVSD as an LVEF < 50%, as in a recent study (10), to discriminate atrial functional MR in the present study. Third, we did not assess LV diastolic function in the present study. Therefore, LV diastolic dysfunction may have been present in some of our study patients. Finally, the generalizability of our findings may be limited by the single-center, retrospective, observational approach.

Catheter ablation is a valid option for the treatment of AF in patients with functional MR and LVSD, even though multiple procedures may be required. The duration of AF, eGFR, and previous stroke can identify patients with function MR with worse results of AF ablation. Freedom from ATa is associated with a reduction in severity of MR and positive LA and LV remodeling in patients with LVSD.

## Data Availability Statement

The original contributions presented in the study are included in the article/supplementary material, further inquiries can be directed to the corresponding author/s.

## Ethics Statement

The study complied with the Declaration of Helsinki and the study protocol was approved by the Research and Development Department at the Royal Brompton and Harefield NHS Foundation Trust.

## Author Contributions

J-TW and JZ designed this study and wrote the manuscript. JJ, RS, ZC, and VM were in charge of the statistical analysis and interpretation of the results. HY, BV, CE, KN, SH, DJ, and HW conducted this study and collected data. TW designed this study and made critical revisions of the manuscript. All authors contributed to the article and approved the submitted version.

## Conflict of Interest

The authors declare that the research was conducted in the absence of any commercial or financial relationships that could be construed as a potential conflict of interest.
